# Optimizing repair of tendon ruptures and chronic tendinopathies: Integrating the use of biomarkers with biological interventions to improve patient outcomes and clinical trial design

**DOI:** 10.3389/fspor.2022.1081129

**Published:** 2023-01-06

**Authors:** David A. Hart, Aisha S. Ahmed, Paul Ackermann

**Affiliations:** ^1^ Department of Surgery, Faculty of Kinesiology, McCaig Institute for Bone and Joint Health, University of Calgary, Calgary, AB, Canada; ^2^ Department of Molecular Medicine and Surgery, Karolinska Institutet, Stockholm, Sweden

**Keywords:** tendon injuries, tendon rupture, biomarkers, improved outcomes, clinical trial design, prognostic biomarkers

## Abstract

Tendons are dense connective tissues of the musculoskeletal system that link bones with muscles to foster mobility. They have complex structures and exist in varying biomechanical, metabolic and biological environments. In addition, tendon composition and mechanical properties can change over the lifespan as an individual ages. Many tendons function in high stress conditions with a low vascular and neuronal supply, conditions often leading to development of chronic tendinopathies, and in some cases, overt rupture of the tissues. Given their essential nature for human mobility and navigation through the environment, the effective repair and regeneration of different tendons after injury or damage is critical for quality of life, and for elite athletes, the return to sport participation at a high level. However, for mainly unknown reasons, the outcomes following injury are not always successful and lead to functional compromise and risk for re-injury. Thus, there is a need to identify those patients who are at risk for developing tendon problems, as well those at risk for poor outcomes after injury and to design interventions to improve outcomes after injury or rupture to specific tendons. This review will discuss recent advances in the identification of biomarkers prognostic for successful and less successful outcomes after tendon injury, and the mechanistic implications of such biomarkers, as well as the potential for specific biologic interventions to enhance outcomes to improve both quality of life and a return to participation in sports. In addition, the implication of these biomarkers for clinical trial design is discussed, as is the issue of whether such biomarkers for successful healing of one tendon can be extended to all tendons or are valid only for tendons in specific biomechanical and biological environments. As maintaining an active lifestyle is critical for health, the successful implementation of these advances will benefit the large number of individuals at risk.

## Introduction

### Purpose of this review

The purpose of this review is to discuss recent advances in the use of biologics and cell therapies in the treatment of injured tendons and developments in the field of biomarkers regarding the healing of injured tendons with good vs. poor outcomes. Subsequently, a discussion regarding the integration of these advances to improve clinical trial design is undertaken. Finally, several issues regarding the generality of extending findings from biomarkers developed regarding one tendon to all tendons is addressed to help focus studies going forward.

### Background

Tendons are complex connective tissues that connect muscles to bones to allow for mobility of joints. They consist of a myotendinous junction (MTJ), a mid-substance, and a specialized enthesis which links the tendon to bone. Thus, tendons exhibit location-specific features (1).

The tissues also consist of the tendon proper plus a distinct surface layer of cells and extracellular matrix (ECM) called the epitenon or paratenon (2). Tendons exist and function in diverse biomechanical environments and as such exhibit both different characteristics as well as common features. Tendons are also dynamic in that they can change their characteristics or properties during aging, such as their stiffness (3–5), and thus their risk for injury and potential loss of function is not uniform across the lifespan. Thus, as the stiffness of tendons such as the Achilles tendon increase with age, the incidence of Achilles tendon ruptures increases, often in males >40 years of age. Thus, the combination of biomechanical, metabolic and biologic environments, and aging could lead to age-dependent risk for the rupture of specific tendons.

Tendons consist mainly of collagen, particularly collagen type 1, plus a number of other minor collagen types and proteoglycans (2). At birth, tendons are very cellular, but gradually become less cellular as matrix is deposited during use and during growth and maturation (6–9), reaching relative homeostasis at skeletal maturity. The intrinsic cells (i.e., tenocytes) of the tendon proper synthesize the extracellular matrix (ECM) which is then laid down in an organized manner using the template established during development, but the surface layers of tendons also contain some vascularity (10), innervation (11–13), and tissue localized cells of the immune system such as mast cells (14). Much of the innervation and microvascularity is mainly associated with the paratenon/epitenon structures surrounding the tendon proper and there is minimal involvement or inclusion of these elements in the tendon proper. The cells of the paratenon/epitennon and the tendon proper appear to be different in some respects (collagen types expressed by the fibroblasts, endothelial cells, neural elements, mast cells), and thus may have different roles in responses after injury during the deposition of early scar tissue (15). Tendons also appear to be affected by sex hormones which likely contribute to sex differences in risk for tendon dysfunction (16, 17) in some tendons but not all (18, 19). Sex also appears to play a role in outcomes after injury to the Achilles tendon in both rats (20) and humans (21). In the rat model, female, male and ovariectomized (OVX) females were assessed after injury and the females differed biomechanically from the males and the OVX animals also differed mechanically and biologically from the females. In the human populations, female surgical patients had more symptoms than male surgical patients following surgery and had lower scores in the heel-rise test at 6- and 12-months post-surgery. However, the majority of patients were male (152 M vs. 30 F) and the age range spanned the onset of menopause. As such, regulation of tendon metabolism in females may also be altered after menopause (22) and affect injury risk, outcomes, and responsiveness to interventions.

Thus, tendons are heterogeneous and appear to develop, mature and adapt to perform functions in unique biological and biomechanical environments. Thus, the tenocytes in the tendon proper respond to the biomechanical and biological environment to allow the tissue to function optimally. They are also dynamic in that they can be influenced by exercise, and their properties and composition can change during the aging process. In addition, they exhibit sex-dependent characteristics (20–22). Taken together, even though they are discussed as a class of tissues, they are very heterogenous, a fact that can complicate the development of effective treatment modalities to enhance repair and regeneration after injury. Furthermore, the low cell density and paucity of vascularity and innervation likely also contribute to an ineffective and prolonged repair response, particularly during aging. However, humans are very heterogeneous, and some patients may have the ability to initiate better repair responses following a tendon injury than others [(23), Chen et al., unpublished].

To define a good vs. a poor outcome in Achilles tendon healing, the literature usually uses the Achilles Tendon Total Rupture Score (ATRS) and the Foot and Ankle Outcome Score (FAOS), both validated questionnaires (23), and often a functional outcome test such as the heel-rise test (HRT) (23). The questionnaires have multiple subscales for scoring, and a lower score indicates a poorer outcome. For example, the ATRS consists of 10 items and a maximal score of 100, indicating full function and no pain. The questionnaires can be administered at any times post-surgery but in the report by Chen et al. (23), the data were obtained at 1-year post-surgery. Good outcomes based on the questionnaires is a score of >80 for each questionnaire, and a poor outcome is a score of <79. The HRT outcomes are related to repetitions of heel raises and limb symmetry (i.e., differences in the functional test outcomes between the injured and contralateral limb). For the paper by Chen et al. (23), the patients with good outcomes had a mean score of 93 and those with a poor outcome had a mean score of 64 for the ATRS so there was a wide separation between the scores for the good and poor outcomes (23).

## Biologics and tendon repair

### Introduction

The need for tendon repair or regeneration can occur after overt rupture of the tissue, partial tearing of the tissue, or due to development of a chronic condition termed tendinopathy, with the latter usually presenting as pain (24). The term tendinopathy covers what used to be termed tendinitis or tendinosis as it more accurately implies a loss of tendon integrity and does not infer any specific mechanisms (inflammation in tendinitis and degeneration in tendinosis). The involvement of inflammatory cells, as might be expected from the term tendinitis, in tendinopathies is quite variable based on the literature as reviewed recently by Jomaa et al. (25). However, conclusions regarding the role of inflammation and inflammatory cells may depend in part on when in the process one investigates, and what type of inflammatory cells one can detect (14).

Biologics can refer to a variety of intervention modalities that range from growth factors, platelet-rich plasma (PRP), mesenchymal stem/stromal cells from different tissue sources, tissue-engineered constructs, to gene therapy approaches. While several of these approaches have obtained some success, albeit often limited, in the treatment of some tendon disorders and often in preclinical model systems, many of the interventions are still experimental in nature and not approved by regulatory bodies. This lack of general success can likely be attributed in part, to having unrealistic expectations and not optimizing the conditions under which they are being applied (26, 27). Furthermore, the heterogeneous types of human tendon injuries, as well as any underlying tendinopathy render much more complex pathologies than those created in preclinical model systems.

### Biologics and tendinopathies

Tendinopathies are usually noticed as the onset of pain either when engaging in an activity, or even at rest (28). Thus, the development of pain is the usual first indication that there has been a change to the integrity of the tissue at some location within the complexity of the tissue (insertion of the tendon into bone/enthesis, mid-substance, myotendinous junction) and surrounding tissues (paratenon, epitenon). As the tendon proper is poorly innervated (11–13) but some of the surrounding tissues are innervated, the pain appears to arise secondarily to the loss of tendon integrity.

As some spring-like tendons such as the Achilles tendon, vs. positional tendons, function normally in a high-stress environments (29), loss of tendon integrity may arise from “overuse” (30–32). Over-use can result from the continued use of the tissue at the high end of its stress-strain curve without adequate time to repair any microdamage that can occur, or it may arise due to age-related changes that influence the properties of the tendon (33). Age dependent changes include alterations to the integrity of the collagen organization in the tendons, alterations in abilities to repair, and biomechanical properties (33). As many elderly individuals try to maintain their activity, the age-related changes may contribute to the increased risk for tendinopathies. Tendinopathies can also be accompanied by altered expression of structural matrix proteins (34) or growth factors (35, 36). Additional risk for development of tendinopathy may also have a genetic basis but this is still an emerging field (37, 38). In some sports such as volleyball where there is considerable jumping and hitting at angles that put abnormal stresses on both lower and upper extremity tendons, one could experience both types of risk, genetics and overuse. As tendons exist in a variety of biomechanical environments with corresponding variation in structural and functional features, the induction of a tendinopathy in a specific tissue (i.e Achilles tendon, patellar tendon, supraspinatus tendon) may exhibit unique induction features and unique response patterns that are tendon-specific. As discussed above, tendons are poorly innervated and vascularized, conditions which specifically under repetitive stress may lead to an inadequate supply of essential factors. Thus, scarce provision of essential healing factors together with endothelial dysfunction may propagate the development of tendinopathy [reviewed in (39)].

The usual course of events following presentation with a tendinopathy is rest and possibly bracing, physiotherapy protocols (40–43), anti-inflammatories (including glucocorticoid injections) (44, 45) (but this is controversial), and finally surgeries to debride the areas around the tendon of abnormal tissue accumulation (46). For some tendons such as the supraspinatus tendon of the shoulder, the tendinopathy may be accompanied by a partial tear, often at the enthesis (47, 48) which may require surgical repair. Unfortunately, while many of these approaches to resolve tendinopathies may offer short term relief, due to the relatively modest repair capabilities of tendons to heal, the outcomes are not fully successful and patients are left with some compromise and for elite athletes, they cannot return to play at the same high level. For some patients, the outcomes after surgery can also be affected by extrinsic factors such as metabolic factors (49) and smoking (50), an extrinsic variable that can also affect the microvasculature. Interestingly, genomics may also play a role in the treatment and management of tendinopathies such as Achilles tendinopathy (37), but again this is an emerging field. One cellular intervention that has been used extensively in the treatment of tendinopathies associated with a variety of tendon is Platelet-rich Plasma (PRP) (51–53). The PRP is usually generated from autologous blood, but it can vary in its preparation leading to variations in perceived effectiveness (reviewed recently in (54); discussed in (55)). As platelets contain many anabolic molecules such as growth factors, their presumed effectiveness in some patients is likely due to enhancing the anabolic environment after injection to the site of interest. It may also exert an anti-inflammatory effect and influence pain [reviewed in (54)]. Some of the variation in PRP preparations can also come from the use of autologous blood as the number and content of platelets can vary with age and sex (56–58). For premenopausal females, PRP “quality” could also be potentially influenced by phase of the menstrual cycle. Based on the variation in effectiveness, some reports have concluded PRP should not be used for some tendinopathies (59), while others report that some patients respond to PRP and others do not (60). Why some patients respond to the PRP, and others do not, is not well defined, but based on the literature it could be due to the PRP preparations, the stage of the injury, the genetics of the patient or other unknown factors!

Another cell therapy that has been used in the treatment of tendinopathies is the use of mesenchymal stem/stromal cells (MSC) from a variety of tissue sources including bone marrow, adipose tissue, and other sources including both allogeneic as well as autologous sources. Such studies have been recently reviewed extensively (61–67). As reviewed by Di Matteo et al. (61) and Mirghaderi et al. (65), often such MSC preparations are used in conjunction with PRP. However, similar to studies with PRP alone, outcomes of studies with MSC or MSC + PRP were often quite variable with some showing improved outcomes while others exhibited no significant improvement in outcomes. This variability in outcomes could be the result of variation in the quality of the cells used, the state of the injury, or the environment (i.e., inflammatory) that the cells are placed (26, 27). Thus, placing the MSC and/or PRP into a catabolic environment could compromise their potential to enhance repair (27).

While the use of MSC has yielded variable results, an emerging field of study regarding tendinopathy treatment has advocated using exosomes or extracellular vesicles (EV) derived from MSC and related cells. These EV or exosomes are shed or released from cells and can contain a variety of growth regulating molecules including proteins and miRNAs (68–71). The contents of such EV or exosome can be influenced by the culture conditions of the cells of origin (72, 73). EV would have to be taken up by endogenous cells and exert an anabolic effect to foster repair of an injured tendon, or perhaps modify cells contributing to a catabolic environment such as macrophages (69).

While all of the cellular therapies discussed above indicate that these approaches may have merit, there is still much that is needed to be done to better understand the variability in outcomes, and certainly more clinical trials are needed as has been suggested by several authors (61, 65, 74) to enhance the quality of evidence for their use.

### Biologics and repair of tendon ruptures

In addition to chronic conditions such as tendinopathies, tendons can undergo traumatic ruptures. Partial or complete tears of tendons such as the Achilles, patellar and rotator cuff tendons can result from participation in sports but are rare. Some tendons such as the Achilles tendon can also undergo rupture following treatment with some antibiotics (75, 76) or statins (77–79) but this again is a rare event and some aspects have been called into question (80–82). Partial tears of the rotator cuff tendon often occur very near the enthesis of the tendon-bone interface, a site very difficult to repair and have a high rate of re-injury (83–85), likely as the enthesis is a unique structure (86, 87). As tendons can have three sites for incurring a tear (myotendinous junction, mid-substance, or the insertion into bone), this further complicates the repair process. Healing of other tendons such as the flexor tendons of the hand is further complicated by the formation of adhesions between the healing tendon and its sheath which compromises function (88). Thus, improvements to healing of flexor tendon ruptures requires better repair tissue formation and reduced adhesion formation (89–91).

Endogenous repair following either conservative care (no surgery) or following surgery leads to scar formation with the risk for functional compromise due to the inferior biochemical and biomechanical properties of the scar tissue (84, 85, 92). However, recently Chen et al. (23, 93) have reported that some patients who have had their Achilles tendon ruptures surgically repaired had very good functional outcomes by 1-year post-surgery while others had less optimal outcomes. Therefore, outcomes following tendon rupture can be variable, possibly due to the quality of the product of the healing process. The basis for a “good vs. poor” healing outcome is not known, but could result from the age, sex, genetics and epigenetics of the individual patient.

While such variation in endogenous outcomes exists, there is a need to improve on the outcomes for many patients, and thus many studies have turned to the evaluation of biological interventions such as those discussed previously (i.e., PRP, MSC, EV), as well as others such as the use of individual growth factors (94–97), or even pulsed electromagnetic fields (98). Much of this effort has been focused on preclinical models thus far in an attempt to better understand the optimal parameters (99–103), but a number of clinical trials are evaluating the potential of the use of biologics to enhance healing outcomes. These studies include the use of PRP (104), the potential of stem cells (105–108), and more recently, the use of EV or exosomes (109–111).

Presently, research on the use of biologics in tendon rupture is progressing but the outcomes thus far have not definitely shown consistent advances to improve healing with enhanced functional outcomes. In part, this may again reside with the fact that there is variability in the effectiveness of endogenous outcomes and therefore, a randomized control trial of a biologic intervention is challenged to show efficacy with regard to a specific intervention.

## Biomarkers of tendon repair

### Biomarkers and Achilles tendon healing

Following rupture of the human Achilles tendon, a number of reports have indicated that after a common surgical repair, endogenous repair without any interventions leads to better outcomes in some patients compared to others (23, 93, 112–114). Thus, there is heterogeneity in functional outcomes at 1-year following surgical repair. Such findings indicate that this variation could be due to genetic or epigenetic heterogeneity in the patient population as other factors such as sex or body mass index do not play an obvious role in the different outcomes (23, 93). A further implication of such findings is that depending on the relative contributions of the number of patients with the potential for good vs. poor outcomes in a clinical trial, the effectiveness of the intervention to be tested may be compromised due to such innate variation in healing. Therefore, it would be advantageous to know which patients are destined for a good vs. poor outcome to be able to concentrate interventions on those with risk for poor outcomes rather than a mix of patients. Thus, having validated biomarkers early after injury that are prognostic of outcome would be valuable for future studies.

Biomarkers that correlate with outcomes following surgical repair of Achilles tendon rupture in patients have been reported (23, 93, 112–114) using tissue samples taken at the time of surgery or *via* analysis of microdialysates obtained from both the surgically repair tendon and the contralateral tendon at 2 weeks post-surgery (23). Depending on the analysis used to identify the biomarker, different molecules have been identified. Based on qPCR assessment of mRNA levels, FGF was identified as a biomarker (93). Using metabolomic approaches, pyruvate and lactate levels were determined to be prognostic biomarkers of healing (113, 114). Subsequent proteomic analysis using mass spectrometry and gene set enrichment and meso scale discovery tools, complement factor D was also identified as a predictor of patient outcomes (23). Presently, these studies are continuing using diverse bioinformatic approaches with proteomic data to identify additional prognostic biomarkers using tissue samples taken at the time of surgery and to further characterize the basis for the differences in outcomes between patient subsets. These additional studies have identified elongation factor-2 and inter-alpha-trypsin inhibitor heavy chain-4 as also being prognostic biomarkers (unpublished observations).

Thus, a cadre of prognostic biomarkers associated with good healing outcomes in patients with a ruptured Achilles tendon have been identified and taken together, are starting to provide a framework to pursue a better understanding of the molecular and cellular basis for good vs. poor outcomes after surgery. However, it remains to be determined whether the same or different biomarkers are also valid as prognostic of healing outcomes after injury to other tendons such as the patellar tendon or the rotator cuff tendon which exist in different biological and biomechanical environments (26). However, if the basis for a good outcome is associated with genetics, then one might expect that some of the biomarkers identified would be in common in different healing environments, particularly those biomarkers related to inflammatory processes. Interestingly, there is some gene expression data available that is correlated with poor outcomes after rotator cuff repair (115). While the number of patients was not large in that study, the authors did find using gene set analysis that those with higher expression of matrix molecules had poorer outcomes post-surgery and that those with higher levels of M1 macrophages did better than those with lower levels. Thus, extracellular matrix gene expression levels and cells associated with inflammation (i.e., marcrophage subsets) were associated with the different outcomes. These findings are somewhat in agreement with the general findings regarding Achilles tendon healing although the analysis for Achilles tendon healing is more detailed presently.

Additionally, just as there appear to be genetic markers for good outcomes after rupture of tendons such as the Achilles and rotator cuff tendons, genetic risk for tearing/rupturing such tendons has also been reported (116–123). Thus, the biomarkers that are proteins result from gene expression (to variable levels dependent on regulation at the DNA level) while genetic risk for injury is in the genome (DNA and potentially epigenetically modified DNA) but how such genomic risk is translated to risk at the tissue level is still largely unknown. While some of these studies are based on risk in twins (123), others have indicated that variations in specific molecules such as MMP-3 (122), iNOS (119), or extracellular matrix genes such as COL1A1 (117) pose risk. Ultimately, there will be a need to integrate the risk factors for injury with those for good and poor healing outcomes to obtain a more complete picture of the landscape in individual patients. However, while rapid genetic testing for risk factors can be performed on DNA from cells from any tissue (white blood cells, skin, mouth epithelial cells), whether such risk factors are modified in specific tissues such as the Achilles tendon *via* epigenetic mechanisms can only be determined using DNA from that tissue, a situation that is problematic from a clinical perspective. Thus, integration of prognostic biomarkers with injury risk in a specific tendon may require evaluation using AI or ML approaches and large numbers of patients.

### Early biomarkers of good healing outcomes are focused on inflammatory processes

It should also be mentioned that an overt injury, such as the rupture of the Achilles tendon is an inflammation-inducing event, and that the surgery to repair such ruptures is another inflammation-inducing event. While chronic inflammation may be detrimental to effective healing, acute inflammation may be viewed as a positive as it is required to remove damaged tissues and initiate the first phase of healing. This perspective is relevant to the current discussion in that several of the biomarkers previously discussed that were identified as being prognostic of a good outcome after surgery were obtained using tissue taken from the ruptured tendon at the time of surgery (93, 115) and thus early (2–4 days post-rupture), or at 2 weeks post-surgery (23, 112–114). Thus, such biomarkers were assessed early in the inflammatory/healing phase, and during the later phase of the inflammatory phase of healing, respectively. This means that prognosis of outcome can be defined very early in the healing process and is likely shaped by the quality and extent of the inflammatory process! This conclusion is also supported by the studies of Blomgran et al. (124) who reported that administration of the anti- inflammatory steroid dexamethasone very early (<4 days post-rupture) after Achilles tendon rupture in a rat model resulted in poor outcomes while administration of the same drug later (5–9 days post-rupture) during the late inflammatory/early remodeling phase led to significantly enhanced/improved outcomes. Thus, good vs. poor outcomes at 1-year in patients may be defined by very early inflammation-related events occurring shortly after the time of injury and in the early phases of healing.

### Immune cell involvement during early biomarker expression

The immune system is involved in healing (125), and as such specific cells of the inflammatory response could shape the initial response leading to good vs. poor outcomes. Two critical cells may be macrophages (125) and mast cells (126). Mast cells are present in tendons such as the patellar tendon (14) and mast cell degranulation occurs during healing of the healing of the rat Achilles tendon after rupture (127), as well as the human tendon [unpublished observations]. Trends for elevated levels of mast cell tryptase, a marker for mast cell degranulation have been detected by proteomic approaches two weeks post-surgery to repair human ATR [unpublished observations]. Mast cell numbers are also reported to increase during the healing of the rabbit flexor tendon after rupture (128). Conversely, the use of mast cell stabilizers, or drugs that prevent mast cell degranulation have been used to modify healing of patellar tendons in mice (Sodium cromolyn) (129), to inhibit abnormal fibrosis leading to joint contractures in rabbits (ketotifen) (130, 131), and to inhibit excessive contraction and fibrosis of skin wound healing in red Duroc pigs (ketotifen) (132). The latter is a genetic model of abnormal dermal wound healing, but it is not known whether similar genetic variation in humans could relate to inflammation-associated healing outcomes. However, such preclinical studies have led to the hypothesis of a myofibroblast-mast cell-neuropeptide axis that contributes to abnormal fibrosis and wound healing (133), a hypothesis also consistent with the findings of Alim et al. (127). In addition, use of the leukotriene inhibitor montelukast (Singular; a drug used to treat asthma) in a rat Achilles tendon injury model very early after injury led to declines in tissue healing parameters (134). As montelukast is a modulator of inflammation, and also a modulator of the PPAR pathway, this may additionally imply that the detrimental down regulation of inflammation very early after injury involves the anti-inflammatory effects of the PPAR pathway as well [discussed in (135)]. Therefore, the use of a drug such as ketotifen, a drug approved for use in humans for >30 years, or montelukast and/or dexamethasone after the initial inflammatory phase of healing could potentially improve healing outcomes on their own or set the stage for improved outcomes with the subsequent use of cell therapies after overt tendon injuries requiring surgery. Thus, the identification of early biomarkers of good outcomes after tendon injury has focused on the inflammatory phase as being a critical early step, and further analysis of why and how these biomarkers relate to specific molecular pathways should be a fruitful direction to focus research efforts going forward.

## Implications of finding biomarkers prognostic of healing outcomes on study design and clinical trials

The “gold standard” for clinical trials is often touted to be the “double blind, placebo-controlled trial” or the “double blind, standard of care-controlled trial”. Even if the subject population is somewhat selected via inclusion and exclusion criteria, human heterogeneity often complicates the ability to form conclusions with a high level of evidence. As discussed in previous sections of this review, this complication has often inhibited the development of good evidence for the effectiveness of biological therapies to improve outcomes after development of tendon disorders. If indeed, genetic and perhaps epigenetic factors are involved in good vs. poor outcomes via endogenous healing, assessing additional interventions to improve healing outcomes would potentially be compromised. This assumes that the biological or cellular interventions would not be able to further improve the outcomes for those having good outcomes based on endogenous healing. If that is in fact true, then one might want to focus the trial of a biological intervention on the population at risk of a poor outcome rather than a mixed population.

A further implication of such biomarkers, and the elucidation of how the molecules influence the healing process, may also relate to whether one should use an autologous or allogeneic biological interventions. Thus, the autologous materials may be compromised by the genetic influences leading to a poor outcome! Since the effectiveness of an autologous biological intervention such as PRP or stem/stromal cell preparations depends on both the quality of what is administered as well as the environment into they are placed, the use of such autologous reagents may compromise the outcomes even if the local environment is controlled (26, 27).

Therefore, in such circumstances, the use of allogeneic materials which do have some risk but are likely not very immunogenic (136, 137), may be preferred. However, extracellular vesicles from stem cells may be even better choices as they have very low immunogenicity and also have immunomodulatory potential (138) so may affect inflammatory environments.

The finding of prognostic biomarkers for healing outcomes for the Achilles tendon, and to a lesser extent the rotator cuff tendon, also has implications for study design in other tendon injury or other connective tissue injury circumstances even if biomarkers are not available for that specific situation. That is, given the heterogeneity observed to date for endogenous healing outcomes, going forward it is likely that each patient in a cohort should be assessed individually rather than combined into groups comparing interventions such as biologic and cell interventions that use autologous or allogeneic reagents. The design of trials may in the future become more precise based on rapid genetic testing of patients for specific markers of outcomes early after injury or as part of a routine health/disease-related DNA platform to assess risk for injury which would aid both trial design to assess risk and need, but this will likely not be achieved in the near future, but it certainly could be a goal. However, such an individual database may need to be updated at intervals as it would not capture the ever-changing epigenetic landscape which could impact the phenotype of the individual patient.

In summary, the finding that some patients heal much better after rupture of a tendon without any interventions than other patients have implications at several levels and some of these are listed:

1. Using validated biomarkers corelated with healing outcomes, interventions to improve healing should focus on populations of patients at risk for poor outcomes to assess efficacy.
2. If such validated biomarkers are not available, large databases of genetic test results may suffice until validated biomarkers are available and can be integrated with genetic databases.
3. When considering cellular therapies to improve healing in those destined for a poor outcome, optimizing the *in vivo* environment they will be placed in is required, as well as assessment of the quality of autologous reagents. If quality is compromised by age, co-morbidities or other factors, then well characterized allogeneic materials, such as extracellular vesicles from mesenchymal stem cells should be considered.

## Developing the use of biomarkers for assessing healing outcomes of tendons and ligaments going forward

### Applications in tendon healing

While the use of biomarkers to identify those patients who will have a good outcome vs. a less good outcome following an Achilles tendon rupture is very promising, the findings thus far also provide the opportunity to explore their development in other circumstances regarding healing of Achilles tendon injuries, as well as other tendon injuries. As the use of cellular therapies become more established, and hopefully assume clinical validation and regulatory body approval, the combined use of such modalities (139–142), the combination of biomarker identification and optimized cellular therapies should lead to significant impact on improved healing. Some of these possibilities are discussed briefly below.

Healing of connective tissue injuries become less effective as an individual ages (143). As individuals are living longer, this means that tendon injuries in those >60 years of age may start to heal with poorer outcomes. For females, this could likely be further influenced by menopause which can affect a large number of physiological systems [discussed in (144)]. Thus, for the female population it would be of interest to ascertain whether the same biomarkers of healing outcome were evident after tendon ruptures in those <40 and those >60. If the biomarkers related to outcomes change significantly with aging in females, then one could ask questions related to why and how, as well as potentially altering the use of autologous vs. allogeneic cell therapies. Similar considerations may be applied to young and old males.

The issue of re-rupture is also one of concern following rupture of a tendon such as the Achilles tendon (145, 146), as well as others (147). Re-rupture in younger patients with Achilles ruptures (145) could be due in part to excessive use of the repaired tendon and more return to play in athletics. However, it would be of interest to assess whether the same biomarkers as were determined by Chen et al. (23) were again indicative of good vs. poor outcomes since the environment would be somewhat different in the second rupture.

More patients have tendinopathy than experience rupture of a tendon such as the Achilles tendon. Those that have Achilles tendinopathy do have an elevated risk for subsequent tendon rupture (128, 148). Other patients rupture their Achilles tendon without evidence for chronic tendinopathy. Therefore, it would be of interest to assess whether biomarkers for good and poor outcomes were the same or different for those with and without tendinopathy. Such information would also potentially inform the effective use of PRP and/or stem/stromal cell interventions.

Thus, the above future research, and likely others will add to the use of appropriate biomarkers with outcomes for tendons, and potentially, start to better define whether some of these biomarkers are surrogates for the different outcomes, or whether they are integral for specific pathways of healing.

### Applications in ligament healing

While ligaments and tendons function in different environments and are functionally different (tendons are the interface between muscles and bone regarding movement and ligaments are mainly stabilizing structures) they do share some structural similarities and respond to injury similarly. However, some ligaments such as the medial collateral ligament (MCL) of the knee mainly functions in the toe region of the stress-strain curve and thus even if it heals “poorly” it can still provide function even if treated non-surgically. In contrast, the ACL functions in a fairly high stress environment in the knee and thus even a partial rupture heals poorly and can compromise function as shown in a sheep model (149). In contrast, a full tear of the ACL, with rupture of one end from the femur can adhere back to another structure but is non-functional (150), or ruptures and cannot adhere back to the original site of attachment and requires reconstruction with an autologous tendon, a cadevaric bone-ACL-bone construct, or some other construct. The reconstruction with an autologous tendon (i.e., a strip of the patellar tendon, hamstring tendon, etc} requires drilling a hole in the femur and the tibia to run the tendon graft through and then anchoring the graft into the bone. Attempts to enhance the integration and healing of the tendon-bone materials with cellular therapies has been attempted (151–153) but the clinical results are still a “work in progress” (151, 154). Over time, some of these ACL reconstructions stretch out/creep (become more scar-like) and become less functional. Whether cellular therapies could prevent such changes from happening could not be found in the literature. In addition, whether biomarkers for this process of becoming a scar could be identified also could not be found in the literature. Therefore, there is still considerable research that is required to address the need to improve healing in ligaments, the Achilles tendon, supraspinatus tendon, and others.

### Methodologic advances

While the proteomic or metabolomic approaches have provided tools to identify biomarkers for good vs. poor outcomes after Achilles tendon rupture, and thus identifying those patients destined for poor outcomes and as such candidates for specific interventions such as cellular therapies or combined cellular/drug therapies, these methodologic approaches are complex and take time to do the analysis. While some aspects of the time frame to obtain prognostic results may be improved, the results thus far would indicate that the “die is cast” very early after injury as to outcomes, so appropriate interventions would have to be initiated quickly after surgery. Perhaps with the identification of a panel of biomarkers for good vs. poor outcomes could be generated to provide very strong predicative value and these could then be the focus of new methodology to rapidly assess them in a complex mixture so as to assist the clinicians involved to then apply the most appropriate interventions to the right patients. Optimally, such assessments could be performed within 1–2 days and then leave time to develop the intervention plan for each patient. As the identification of biomarkers that correlate with outcomes is an emerging field, further strengthening of their use in clinical decision making should make investment in research leading to the above methodologic advances a valuable set of tools.

In addition to the above methodologic advances, it would also be advantageous to have methods to noninvasively monitor the healing of a tendon such as the Achilles tendon at intervals between the time of surgery and out to 1-year post-surgery and beyond. One technique that could be applied to noninvasively assess remodeling and function of the healing Achilles tendon is *via* measuring shear wave speed and properties (155–157). It may be possible to correlate scar remodeling post-surgery and the return of tissue organization that may reflect normal (compared to the contralateral tendon) properties along with physical dimensions. By assessing patients longitudinally, it may also be used to assess the healed tendon of those with good vs. poor outcomes beyond one year to determine whether the healing differences are due to intrinsic differences established at the time of injury or are a rate phenomenon and those with a poor outcome at 1-year may improve by 2- or 3-years post-injury. Such methodologic abilities could provide additional insights into the mechanisms involved. Such insights may reinforce the need to enhance those destined for a poor outcome at 1-year using cellular or combination therapies at the time of surgery.

## Conclusions

Tendons are complex connective tissues that operate in different biomechanical environments and under different individual metabolic influences. As a group, they have been considered to heal poorly due to their relative paucity of innervation and microvascularity. However, more recently it has been found that after surgical repair of Achilles tendon ruptures some patients experience good outcomes, while others experience poor outcomes. The identification of biomarkers for good and poor outcomes regarding healing of this tendon, and to a lesser degree the rotator cuff tendon implies that there is heterogeneity in the healing process. This heterogeneity in healing outcomes, and the characterization of the basis for the heterogeneity should lead to more detailed precision medicine for patients.

This variation in endogenous healing outcomes also has considerable potential implication for why it has been difficult to develop strong evidence for the effectiveness of biological interventions, including cell therapies to improve healing outcomes. Thus, assessment of patients using validated biomarkers of good vs. poor outcome risk will identify those patients that need interventions to augment the healing process, and thus lead to a more accurate assessment of intervention effectiveness in clinical trials. Moreover, validated biomarkers will help to speed up answers and the development of biological interventions in clinical trials. In addition, more complete characterization of patients, including both risk for tendon injury and other connective tissue injuries, will also contribute to more individual patient trial designs in the future. Ultimately, this should lead to better management of patients with connective tissue injuries, and specifically, those with tendon injuries.
